# Divergent Thinking in Parkinsonism: A Case–Control Study

**DOI:** 10.3389/fneur.2017.00534

**Published:** 2017-10-25

**Authors:** Margherita Canesi, Maria Luisa Rusconi, Emanuele Cereda, Alessandra Ranghetti, Viviana Cereda, Federica Moroni, Gianni Pezzoli

**Affiliations:** ^1^Parkinson Institute, Azienda Socio Sanitaria Territoriale Pini-CTO, Milano, Italy; ^2^Department of Human and Social Sciences, Università degli Studi di Bergamo, Bergamo, Italy; ^3^Nutrition and Dietetics Service, Fondazione IRCCS Policlinico San Matteo, Pavia, Italy

**Keywords:** creativity, divergent thinking, parkinsonism, progressive supranuclear palsy, multiple system atrophy, Parkinson’s disease, Torrance test

## Abstract

**Background:**

Creativity is a multidimensional phenomenon and an important component of human capacities. This ability is characterized by the involvement of several cognitive functions particularly linked to the prefrontal cortex. We compared divergent thinking, a measure of creativity, in patients affected by progressive supranuclear palsy (PSP), other parkinsonian syndromes, and healthy controls (HCs).

**Methods:**

Creativity features were evaluated using the Abbreviated Torrance Test for Adults (ATTA). Consecutive PSP outpatients were screened for inclusion. Then, patients with multiple system atrophy (MSA) and Parkinson’s disease (PD) and a group of HC were studied. All groups have preserved cognitive functions and were matched for gender, education, disease duration, and age at onset with exception of PD patients who were matched by disease severity rather than disease duration.

**Results:**

PSP patients were characterized by lower values in total ATTA and all subscales than HC and both MSA and PD patients. No differences were found comparing HC versus both MSA and PD patients. PSP patients were characterized by more impaired frontal functioning [assessed by means of Frontal Assessment Battery (FAB)] than HC and both PD and MSA patients.

**Conclusion:**

In the present study, ATTA was significantly lower in PSP patients than in the other study groups. The worst performance in ATTA-total score and the lower score in FAB in PSP patients support the role of frontal function in creative processes.

## Introduction

Creative skills and creative process are still a fascinating, but unresolved topic up for debate as regards a variety of brain diseases (in particular, stroke and neurodegenerative diseases). It is known as the ability “to understand, develop and express in a systematic, novel orderly relationship” and also “to generate ideas that are both novel and useful in a particular social setting” (1, 2). Guilford assimilated creativity with divergent thinking (DT) which is the ability to generate a number of ideas that are useful to solve particular problems (3).

Measurement of creativity is crucial in order to be able to study and understand the phenomenon. One approach is to measure DT, which includes a number of aspects, such as fluency, flexibility, originality, and elaboration (4). Although DT is not the equivalent of creativity, it has frequently been used as a surrogate measure of creative cognition both in healthy controls (HCs) and different patients, such as those with cerebrovascular disease (5), or neurodegenerative diseases (6, 7). Also neuropsychological examinations are an effective approach for investigating creative skills. They are valid tools to establish which cognitive functions and brain regions are involved. Although creativity is an important component of human capacities, the interest in this field has recently grown and the underlying mechanisms and neural correlates have not been completely elucidated. This ability, expressed in different domains (i.e., arts, technology, and science) is characterized by the involvement of several higher cognitive functions (particularly, executive functions) linked to the prefrontal cortex (PFC): mental-set shifting, inhibitory control, and updating working memory (8).

Nevertheless, inconclusive results link executive functions and creativity. Miller et al. (9) have studied patients with semantic variants of frontotemporal lobar degeneration (FTLD), who created works of art in spite of severe degeneration of striatal, temporal, and left inferior frontal–insular regions. On the other hand, significant deterioration of creative ability was observed in frontal variants of FTLD (10). In addition, heterogeneity in creative thinking has been observed in other neurodegenerative diseases, such as Alzheimer’s disease, Lewy bodies disease, and stroke affecting a number of different brain areas, which may be characterized by variable involvement of the PFC (11). Taken as a whole, these data suggest that the integrity of PFC is associated with creative skills.

In addition, DT—which is affected by mesolimbic dopamine—may be influenced or triggered by the use of psychotropic substances. This has recently been studied in Parkinson’s disease (PD) patients (7–12), in whom dopaminergic drugs seem to be able to enhance verbal and visual creative thinking. However, the observations support the idea that dopaminergic treatment may enhance the drive to create with no significant influence on DT itself (13, 14).

There are also other neurodegenerative diseases that are characterized by different types of cortical and subcortical dysfunction, dysregulation of dopamine circuitries, and response to dopaminergic therapy. Progressive supranuclear palsy (PSP), a predominant 4-repeat tauopathy, is characterized by midbrain atrophy, as well as atrophy of the internal pallidum, subthalamic nucleus, thalamus, and frontal cortex resulting in rigid-akinetic syndrome, ocular motor dysfunction, postural instability and falls, frontal signs, and bulbar dysfunction (15). No therapeutic strategies are available and dopaminergic treatment is almost ineffective. Specific cognitive domains are affected in PSP patients and include behavioral change (apathy, impulsivity, irritability) (16, 17), executive deficits (18, 19), verbal fluency (20, 21), memory (22), and social cognitive deficits (18). Cognitive impairment is evident also in PSP patients not identified as demented by means of Mini-Mental State Examination (MMSE) (19). Multiple system atrophy (MSA), an α-synucleinopathy, is a disorder with combined manifestations of parkinsonism, autonomic failure, and cerebellar ataxia. It is characterized by neuropathological changes involving subcortical (basal ganglia) and cortical structures (23). Symptomatic therapeutic strategies are lacking although mild and temporary responses to dopaminergic stimulation are observed in some cases. Although the presence of cognitive decline has been considered as an exclusion criterion for MSA (24–26), in more recent works, it was found to be present also in the early phase of the disease (18–27). As suggested by neuropsychological studies (18–28), executive dysfunction is the prominent cognitive disturbance in MSA (both parkinsonian and cerebellar variant). Neuroimaging and postmortem findings support the involvement of frontal, temporal and parietal regions in MSA cases (29, 30). Of note, cognitive impairment, particularly executive dysfunction, is prominent in PSP and MSA. Cognitive decline largely overlaps in PSP and MSA and only quantitative differences may clinically discriminate between the two diseases (31). Executive abilities are relevant for creative thought as suggested in the work of Benedek et al. (32). Moreover, executive functions seem to be linked to frontal areas both in functional and structural terms (33). To further explore the involvement of executive functions in creative abilities measured by means of DT, we decided to test patients with PSP in comparison with patients suffering from other parkinsonian diseases (MSA and PD) and with HC.

## Materials and Methods

### Patient/Subject Selection

We conducted a prospective case–control study at the outpatient clinic of the Parkinson Institute (ASST Gaetano Pini-CTO, Milan, Italy). First, consecutive PSP patients were included according to the criteria of the National Institute of Neurological Disorders and Stroke/Society for the diagnosis of Progressive Supranuclear Palsy (NINDS/SPSP) (24). Then, we recruited two additional groups of patients: patients with MSA [matched (1:1) by gender, disease duration (±1 year), age at onset (±1 year), and education (±1 year)] and PD [matched (1:1) by gender, education (±1 year)], age at assessment (±1 year), and disease severity (Hoehn–Yahr stage)] (34). PD patients could not be matched by disease duration due to the more benign course of the disease. Also a group of HC [matched (1:1) by gender, education (±1 year), and age at assessment (±1 year)] was studied.

Probable MSA was diagnosed according to the criteria provided in the second consensus statement on the diagnosis of MSA idiopathic PD was diagnosed in agreement with the UK PD Society Brain Bank criteria (35). All patients underwent magnetic resonance imaging to exclude alternative diagnoses, such as vascular parkinsonism or normal pressure hydrocephalus (36). We excluded patients with severe deficits which precluded the execution of the neuropsycological evaluation, i.e., severe dysarthria, severe bradykinesia, and rigidity with the impossibility of writing and drawing. We also excluded patients on antipsychotic and antidepressant therapy (6). Also patients with a positive history of impulsive–compulsive behavior were excluded (37).

Dementia (as assessed by MMSE score > 24/30 points) (38, 39) was an exclusion criterion for all patients and HC and nobody had been a professional artist or had been creating works of art as a hobby in the past. Patients treated with neurosurgical procedures (mainly deep brain stimulation) were excluded and none of the HC included was a caregiver or family member of study patients.

### Measurements

The Abbreviated Torrance Test for Adults (ATTA) (4) was used to assess creativity. Specifically, the test is used to investigate four factors of creative thinking: *Flexibility* (a kind of thinking that allows an exchange of ideas and strategies, and the ability to pass from one category to another), *Fluency* (the ability to conceive many ideas and hypotheses without focusing on their quality, which is potentially useful in problem solving), *Originality* (the capacity to find unusual and rare answers), and *Elaboration* (the ability to further develop a concept by adding new elements).

A visuospatial task [Clock Drawing Test (CDT)] (40) and a frontal functions battery [Frontal Assessment Battery (FAB)] (41) were also administered. All tests were administered by trained psychologists, expert in movement disorders, in the morning hours, 90 min after the last dose of dopaminergic therapy. Patients who were not undergoing dopaminergic therapy were tested during their best condition.

On the same day, clinical work-up included the evaluation of disease severity and motor functions (in worst “OFF” conditions) by means of the Hoehn and Yahr (H&Y) staging system (34) and the Unified Parkinson Disease Rating Scale (UPDRS) part III (motor score) (42), respectively. Although specific scales for PSP (PSP rating scale) and MSA (UMSARS) are available (43, 44), we used UPDRS part III to measure and compare disability conditions between the different neurodegenerative diseases. Information on dopaminergic treatment was also recorded in all study groups and doses of dopaminergic medication were converted to equivalent levodopa-equivalent daily doses (LEDDs) (45).

### Statistical Analysis

Sample size was calculated on the difference in primary outcome variable (ATTA total score) between PSP patients and HCs. Considering a meaningful difference in total ATTA score of at least 20 points and a common SD of 18 points (13), the sample size sufficient to have a power of 80% with a type I error of 5% is 13 patients in each group.

Continuous variables were reported as mean and SD and compared between groups using the Student’s *t*-test for paired data with exception of the comparison with CBS patients (test for unpaired data). Specifically, we compared PSP patients to HC and other parkinsonian syndromes. The comparison between HC and other types of parkinsonian syndromes was also investigated. Categorical variables were presented as count and percentage. Finally, correlations between test scores were analyzed using Kendall’s tau test.

All data were analyzed using MedCalc Statistical Software version 16.8.4 (MedCalc Software bvba, Ostend, Belgium), setting the level of significance at a two-tailed *P*-value of <0.05.

### Ethics

The study was performed in agreement with the principles of the Declaration of Helsinki and the protocol was approved by the local Ethics Committee (Protocol 272-2010). We obtain written informed consent from every patient and control subject recruited.

## Results

**Figure 1:**
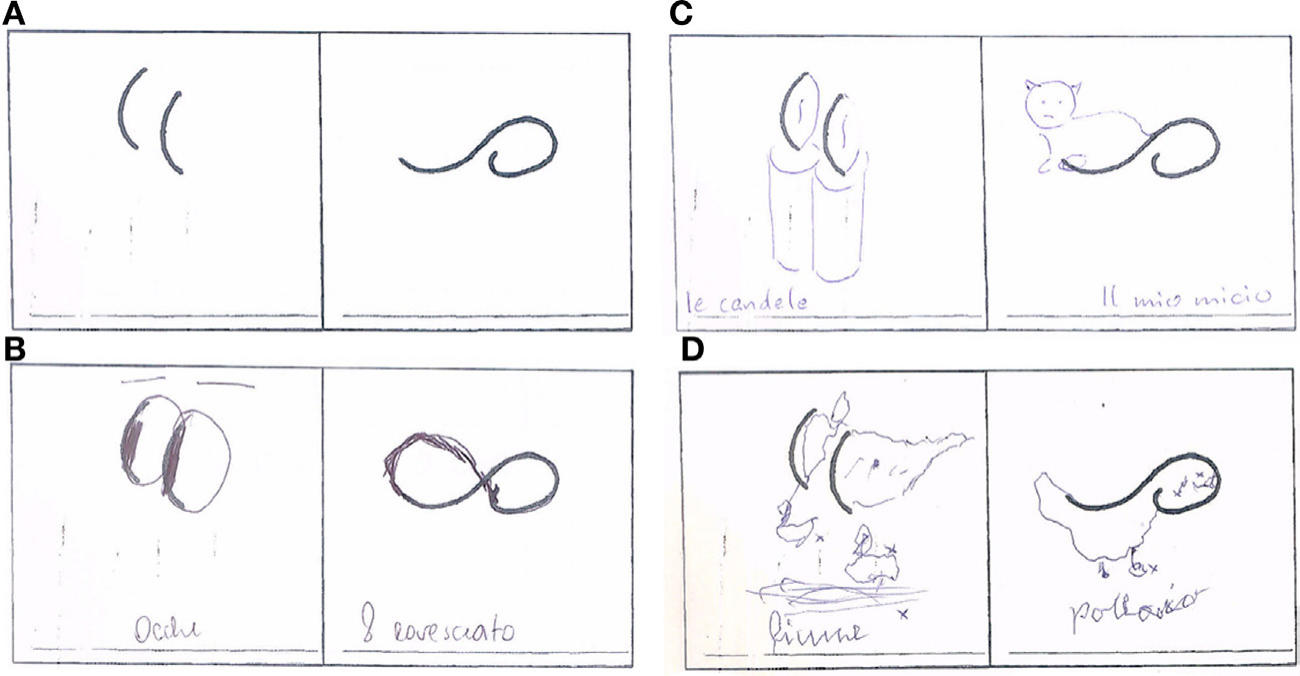
Exemplification of drawing test in the different study groups [**(A)** basic drawing; **(B)** progressive supranuclear palsy; **(C)** Parkinson’s disease; **(D)** multiple system atrophy].

Demographic and clinical characteristics of the study groups are shown in Table 1. As expected, taking into account the less severe progression of PD and the decision to match by disease severity, PD patients were characterized by younger age at onset and longer disease duration. No differences were found in motor disability between PSP and other parkinsonian syndromes. Most PSP, MSA and PD patients were on dopaminergic therapy, mainly levodopa (9, 10, and 13 patients, respectively), while a substantial number of PD patients were receiving a dopamine agonist (7 patients). Results of neuropsychological tests are presented in Table 2. In the absence of cognitive deterioration, as assessed by MMSE, PSP patients were characterized by more impaired frontal functioning (FAB) than HC and both PD and MSA patients. Significantly lower values (*P* < 0.05) in FAB were observed in MSA patients compared to controls. MSA patients showed also lower CDT score than HC (*P* < 0.05). Data regarding creativity, as assessed by ATTA, are reported in Table 3. With the exception of the comparison in fluency score between PSP and MSA, PSP patients were characterized by lower values in total ATTA and all subscales than HC and both MSA and PD patients. No differences were found comparing HC versus both MSA and PD patients. Although the patients included should have had normal cognitive functions, the presence of cognitive alterations could not be fully excluded. We therefore explored the possible relationship between DT and specific cognitive subscores related to functions potentially contributing to creativity: verbal fluency by FAB-lexical fluency subscore and visual-constructional abilities by MMSE-drowing copy subscore. PSP patients performed significantly worse than all the other study groups (Figure 1), (Table 2). In addition, at population level (all groups pooled), ATTA total score was significantly correlated with both FAB-lexical fluency (τ = 0.20; *P* = 0.034) and MMSE-design copy (τ = 0.40; *P* < 0.001).

**Table 1:** Demographic and clinical characteristics of the study groups.

	PSP (*N* = 13)	MSA (*N* = 13)	PD (*N* = 13)	HC (*N* = 13)
Male gender, *N* (%)^a^	7 (53.8)	7 (53.8)	7 (53.8)	7 (53.8)
Education (years), mean (SD)^a^	9.7 (3.8)	10.6 (4.8)	10.5 (5.1)	10.5 (3–6)
Age at assessment (years), mean (SD)^a^	66.7 (4.3)	65.2 (6.2)	65.0 (11.9)	66.0 (6.2)
Disease duration (years), mean (SD)^b^	4.7 (1.7)	4.6 (2.4)	9.6 (6.4)	–
Age at onset (years), mean (SD)	62.0 (4.9)	60.3 (7.3)	55.0 (10.5)	–
UPDRS-III, mean (SD)	42.2 (8.8)	36.8 (9.5)	34.7 (8.8)	–
H&Y stage, mean (SD)^c^	3.5 (0.7)	3.4 (0.6)	3.1 (0.3)	–
LEDDs (mg/die), mean (SD)	292 (262)	533 (472)	468 (257)	–
Levodopa therapy, *N* (%)^a^	8 (61.5)	10 (76.9)	9 (69.2)	–
Dopamine agonist, *N* (%)^a^	1 (7.7)	1 (7.7)	7 (53.8)	–
No dopaminergic therapy, *N* (%)^a^	4 (30.8)	3 (23.1)	1 (7.7)	–

*PSP, progressive supranuclear palsy; MSA, multiple system atrophy; PD, Parkinson’s disease; HC, healthy controls; LEDDs, levodopa equivalent daily dose; H&Y stage, Hoehn and Yahr stage; UPDRS-III, Unified Parkinson Disease Rating Scale part III (motor score)*.

*^a^Common matching variable*.

*^b^Additional matching variable for PSP and MSA patients*.

*^c^Additional matching variable for PD patients*.

**Table 2:** Neuropsychological evaluation.

Test	PSP (*N* = 13)	MSA (*N* = 13)	PD (*N* = 13)	HC (*N* = 13)	*P*-value^a^	*P*-value^b^	*P*-value^c^
MMSE (score), mean (SD)	27.0 (1.3)	26.8 (1.4)	26.9 (2.0)	26.7 (1.1)	0.72	0.90	0.35
MMSE—drawing copy (score), mean (SD)	0.4 (0.5)	0.8 (0.4)	0.9 (0.3)	1.0 (0.0)	0.027	0.003	0.001
FAB—adjusted score, mean (SD)	12.5 (2.4)	14.8 (1.5)	15.0 (2.2)	16.1 (1.1)	0.019	0.022	<0.001
FAB—equivalent score, mean (SD)	0.5 (0.7)	1.9 (1.4)	2.5 (1.5)	3.2 (0.9)	0.009	0.002	<0.001
FAB—lexical fluency, mean (SD)	1.6 (1.0)	2.8 (0.4)	2.5 (0.9)	2.5 (0.5)	0.002	0.049	0.014
CDT (score), mean (SD)	3.3 (1.3)	2.8 (1.2)	3.7 (1.4)	4.2 (1.1)	0.25	0.54	0.08

*MMSE, Mini-Mental State Examination; FAB, Frontal Assessment Battery; CDT, Clock Drawing Test*.

*^a^PSP vs. MSA by paired t-test*.

*^b^PSP vs. PD by paired t-test*.

*^c^PSP vs. HC by paired t-test*.

**Table 3:** Creativity features as assessed by the Abbreviated Torrance Test for Adults (ATTA).

Score	PSP (*N* = 13)	MSA (*N* = 13)	PD (*N* = 13)	HC (*N* = 13)	*P*-value^a^	*P*-value^b^	*P*-value^c^
Fluency, mean (SD)	7.3 (3.6)	9.7 (5.2)	11.5 (3.0)	12.9 (2.4)	0.24	0.005	0.001
Flexibility, mean (SD)	5.1 (1.8)	7.7 (4.3)	9.5 (2.7)	10.5 (2.6)	0.037	<0.001	<0.001
Originality, mean (SD)	5.8 (4.0)	10.5 (6.3)	15.1 (6.0)	14.7 (5.5)	0.016	<0.001	0.001
Elaboration, mean (SD)	8.2 (5.6)	17.2 (15.7)	19.3 (9.5)	18.2 (8.6)	0.031	0.003	0.003
ATTA total, mean (SD)	26.4 (12.6)	45.2 (28.5)	55.4 (18.2)	56.3 (16.8)	0.025	<0.001	<0.001

*^a^PSP vs. MSA by paired t-test*.

*^b^PSP vs. PD by paired t-test*.

*^c^PSP vs. HC by paired t-test*.

## Discussion

In the present study, DT, measured by means of ATTA, was significantly lower in PSP patients than in the other study groups. The worst performance in terms of ATTA-total, along with the lower score in FAB, found in PSP patients suggests that frontal function has an important role in creative processes.

The role of the frontal cortex in DT has been pointed out in previous studies in HC. Regional cerebral blood flow measurements were used in control subjects with high and low scores in creative tests to study the functional organization of the cortex. Highly creative subjects showed bilateral frontal activation during DT tasks (46). Fink et al. (47) found frontal alpha synchronization as a selective top-down inhibition process in HC during DT tasks by means of the analysis of the alpha band of the electroencephalogram. Moreover, creativity requires cognitive abilities linked to prefrontal regions of brain. Dietrich proposed four basic types of creative insight occurring during two modes of thought (deliberate and spontaneous) and two types of information (emotional and cognitive) (48). Transcranial magnetic stimulation over frontal lobes can increase creativity in normal subjects (49). Flaherty suggested a useful and simplified model that helps to characterize the different contributions of the frontal, temporal and limbic systems (6). Trained musicians as creative group were investigated as regards creativity and frontal cortical activity in two experimental sets. In the first experiment, musicians showed convergent and DT and scored higher in the Schizotypal Personality Scale when compared with HC. In the second set, the authors observed greater frontal activation in musicians than HC during DT tasks at near-infrared spectroscopy (50). Although all patients included in our study were non-demented according to MMSE scores (inclusion criteria), significant differences were observed in frontal functions evaluated by means of FAB, a bedside test of executive functions, which includes abstract conceptualization, fluency, sequencing, inhibition and interference with motor command (51). In agreement with a previous study (18), PSP patients performed worse than other study groups. No differences were evident among PD, MSA and HC groups. The most common cognitive deficit in the majority of PSP patients concerns executive function, which has been linked to the involvement of the medial PFC (and the connections with the striatum) (52, 53). Verbal fluency, incorporated in the FAB test, is impaired in PSP patients and enables to discriminate between PSP and PD. Indeed, seven or less P-words per minute suggests PSP (54–56). The severity of executive dysfunction and the negative ATTA outcome observed in PSP group provide further evidence suggesting a relationship between frontal integrity and creative thought.

Clock Drawing Test was significantly worse than MSA when compared to HC, likely due to more impaired visual-spatial abilities (40–57). PD and PSP groups performed worse when compared to HC, but data produced just a trend, no significant differences. Besides, our results support the hypothesis of a lack rather than a weak relationship between ATTA scores and dopaminergic treatment, and point out the importance of the involvement of different brain areas, including the frontal area, in the creative process. It has been previously pointed out that dopaminergic treatment is involved in the emerging of creative drive (7–14). Indeed, no differences emerged in ATTA scores between HC (with no dopaminergic treatment) and both the PD and the MSA (most patients were on dopaminergic therapy) group. On the contrary, significant differences in creative tasks emerged between HC and PSP group, although some PSP patients were on low-dose dopaminergic therapy (69.2%). Nevertheless, owing to the small number of patients included in the study, we cannot entirely exclude any influence of dopaminergic treatment on ATTA between-group comparisons. Therefore, the present study is consistent with previous observations that enhancement in creative activity may occur, mainly in predisposed patients (13). Moreover, the results of this study suggest that ATTA could be used as an additional diagnostic tool to help differentiate PD from PSP.

Although the results are interesting, we are aware that our study has some limitations. First, our sample size was relatively small due to the rare nature of parkinsonisms and the inclusion criteria. Therefore, the study groups included are not fully representative of the patient populations. Particularly, it is known that cognitive deficits are frequent in these patients (57) and we included only patients without global cognitive deficits, with the aim of identifying patients able to understand the tasks. Besides, the study design and the matching procedure enabled us to reduce the bias associated with potential confounders. Second, we performed a brief, non extensive neuropsychological assessment in order to avoid fatigue and attentional problems during tests in patients with parkinsonism. However, based on previous observations, a more detailed evaluation of neuropsychiatric disorders (e.g., by means of neuropsychiatric inventory) would have provided more information on cortical functions (58, 59). Third, we are aware that creativity is not completely attributable to the performance of the frontal cortex, as previous findings have revealed the selective involvement of different brain regions in diverse aspects of creativity (60). Finally, although the use of clinical criteria (e.g., disease duration, response to levodopa) and neuroimaging investigations improved the probability of a correct diagnoses, diagnostic certainty can be achieved only by pathological confirmation on postmortem tissue examination. In conclusion, neurodegenerative diseases, such as PSP, MSA, and PD, are characterized by the involvement of different brain areas and the analysis of creative competence enables us to speculate about the role played by these brain areas. Particularly, PSP patients performed worse than the other patients, despite having disease of similar severity and similar global cognitive performances. These results suggest that creative accomplishment requires most of all the integrity of the frontal areas, which appear to play a prominent, albeit not exclusive role.

## Ethics Statement

Ethics Committee: ASST-G Pini, Centro Specialistico Ortopedico Traumatologico, Milano, Italy. The study was performed in agreement with the principles of the Declaration of Helsinki and the protocol was approved by the local Ethics Committee (Protocol 272-2010). We obtain written informed consent from every patient and control subject recruited.

## Author Contributions

All the authors significantly contributed to the work, read, and approved the final version of the manuscript. MC, MR, AR, and VC contributed to study design. MC, AR, VC, and FM; contributed to data collection. MC, MR, EC, AR, VC, and FM contributed to data analyses and interpretation; contributed to manuscript drafting. MC, MR, EC, AR, VC, and GP contributed to critical revision of the manuscript.

## Conflict of Interest Statement

The authors declare that the research was conducted in the absence of any commercial or financial relationships that could be construed as a potential conflict of interest. The reviewer SM and handling Editor declared their shared affiliation.
